# Conventional and drug‑eluting bead transarterial chemoembolization in patients with inoperable intrahepatic cholangiocarcinoma: a meta‑analysis

**DOI:** 10.20452/wiitm.2024.17906

**Published:** 2024-11-05

**Authors:** Su‑Rong Pan, Xue‑Wen Wo, Hong‑Fang Zhu, Feng‑Fei Xia

**Affiliations:** Department of Gastroenterology, Binzhou People’s Hospital, Binzhou, China; Department of Neurology, Binzhou People’s Hospital, Binzhou, China; Department of Interventional Vascular Surgery, Binzhou People’s Hospital, Binzhou, China

**Keywords:** conventional, drug‑eluting beads, intrahepatic cholangiocarcinoma, transarterial chemoembolization

## Abstract

**INTRODUCTION:**

In patients with inoperable intrahepatic cholangiocarcinoma (ICC), both conventional transarterial chemoembolization (cTACE) and drug‑eluting bead TACE (DEB‑TACE) can be employed as therapeutic interventions. However, the relative advantages of these strategies remain to be clarified.

**AIM:**

This meta‑analysis was performed to compare the safety and efficacy of DEB‑TACE and cTACE in the treatment of ICC.

**MATERIALS AND METHODS:**

A comprehensive search of the Cochrane Library, PubMed, and Wanfang databases was conducted to identify publications that were pertinent to the present meta‑analysis. The primary outcome of interest was the overall survival (OS) rate. Secondary outcomes were progression‑free survival (PFS), disease control rate (DCR), objective response rate (ORR), and adverse event (AE) rate. Heterogeneity was evaluated using the I 2 statistic, while publication bias was assessed with the Egger test.

**RESULTS:**

A total of 6 articles involving 283 and 178 patients who received cTACE and DEB‑TACE treatment, respectively, were included in this study. DEB‑TACE was superior to cTACE in terms of DCR (*P *= 0.004), PFS (*P *<0.001), and OS (*P *= 0.004), despite comparable pooled ORRs. No intergroup differences were observed with respect to AE occurrence. The ORR, DCR, and OS end points showed significant heterogeneity (I2 = 79%, I^2^ = 61%, and I^2^ = 95%, respectively). Additionally, the OS end point was subject to substantial publication bias (Egger test, *P *= 0.002).

**CONCLUSIONS:**

DEB‑TACE was shown to be superior to cTACE with respect to efficacy, while the safety profile of these 2 interventions was similar. Consequently, DEB‑TACE offers additional value in the management of inoperable ICC.

## INTRODUCTION 

Intrahepatic cholangiocarcinoma (ICC) accounts for 10% to 15% of all primary liver cancers, and it is the second most commonly diagnosed primary liver cancer type.^1^^;^^2^^;^^3^ While surgery can cure the disease, less than 30% of individuals with ICC are eligible for such treatment.^4^ What is more, such patients are still at a risk of disease recurrence, resulting in 5‑year overall survival (OS) and disease‑free survival rates of approximately 55% and 41.7%, respectively.^5^

In individuals with inoperable ICC, locoregional therapies are the primary approach to prolonging survival and delaying progression of the disease.^6^^;^^7^^;^^8^Transarterial chemoembolization (TACE) is often employed as a locoregional approach for managing ICC or hepatocellular cancer (HCC).^8^^;^^9^It effectively disrupts blood supply of the tumor while also allowing for sustained local release of therapeutic agents. Conventional TACE (cTACE) and drug‑eluting bead TACE (DEB‑TACE) are 2 available techniques employed

**Table 1:** Modified Response Evaluation Criteria in Solid Tumors

Criterion	Definition
Complete response	Disappearance of any intratumoral arterial enhancement in all target lesions
Partial response	A decrease by at least 30% in the sum of diameters of viable (enhanced in the arterial phase) target lesions, with the baseline sum of the target lesions diameters serving as a reference
Stable disease	Not meeting the criteria of partial response or progressive disease
Not meeting the criteria of partial response or progressive disease	An increase by at least 20% in the sum of the diameters of viable (enhanced) target lesions, with the smallest sum of the viable (enhanced) target lesion diameters recorded since treatment initiation serving as a reference

in this therapeutic setting.^8^^;^^9^ In cTACE, lipiodol serves as both the drug carrier and the embolic agent. Still, the utility of lipiodol is hampered by its highly fluid nature and poor amenability to sustained drug release.^6^DEB‑TACE is an alternative approach that overcomes these limitations, as DEBs facilitate controlled, localized chemical release, thereby increasing the concentrations of drugs within tumors while limiting their systemic spread more effectively than in the case of cTACE.^6^

While DEB‑TACE is used increasingly often to manage HCC,^9^ its utility as a treatment for ICC has been explored to a limited extent. A systematic evaluation was performed to compare cTACE and DEB‑TACE as treatment alternatives for ICC. The results showed that DEB‑TACE was linked with a higher disease control rate (DCR) and superior tumor response outcomes, as compared with cTACE.^6^That review, however, was based on single‑arm studies and was subject to a significant selection bias,^6^ emphasizing the need for a meta‑analysis focused on comparative studies.

## AIM 

The current meta‑analysis was designed to compare the relative safety and efficacy of cTACE and DEB‑TACE as therapeutic approaches to inoperable ICC.

## MATERIALS AND METHODS 

### Study selection 

The present meta‑analysis followed the rules set by the Preferred Reporting Items for Systematic Reviews and Meta‑Analyses (PRISMA) reporting guidelines and was registered at INPLASY.COM (registration No. INPLASY202460085).

A comprehensive search was undertaken to identify relevant papers published up until June 2024 in the PubMed, Cochrane Library, and Wanfang databases, using the following prespecified search terms: ((intrahepatic cholangiocarcinoma) OR (ICC)) AND ((transarterial chemoembolization) OR (TACE)) AND (conventional) AND ((drug‑eluting beads) OR (DEB)).

The inclusion criteria comprised 4 domains:

1. type of intervention: cTACE and DEB‑TACE;
2. population: patients with inoperable ICC;
3. study type: comparative analyses; and
4. language of publication: no limitations.

Case reports, reviews, letters, nonhuman studies, and single‑arm studies were excluded from analysis.

### Data extraction 

Two authors (SRP and XWW) independently chose eligible studies and extracted the relevant data, with a third author (FFX) serving as an arbiter in the case of any disagreements. Data collected from the included studies comprised the primary author name, year of publication, country of origin, study design, patient population, age distribution, sex ratio, tumor size, liver function test findings, number of tumors, and tumor stage. The collected outcome data included progression‑free survival (PFS), OS, objective response rate (ORR), DCR, and adverse event (AE) occurrence.

### Quality evaluation 

For randomized controlled trials (RCTs), quality evaluation was performed with the Cochrane risk‑of‑bias method. This involved assigning a low, high, or unclear bias risk to each of the following categories: performance, reporting, detection, selection, attrition, and other biases.

The Newcastle–Ottawa scale (NOS).^10^ was used to assess observational studies, with 4, 3, and 2 points assigned to the selection, exposure, and comparability criteria, respectively. Studies with NOS scores of 7 or higher were considered to be of good quality.

### End points 

The primary study end point was OS, while PFS, ORR, DCR, and AE incidence served as secondary end points. The analyzed AEs included elevated levels of aspartate aminotransferase (AST) or alanine aminotransferase (ALT), hyperbilirubinemia, and hypoalbuminemia. The patients who showed a complete or partial improvement in their condition were classified as having an objective response. Disease control encompassed both the patients with an objective response and those with stable disease. Evaluation of tumor responses was performed according to the modified Response Evaluation Criteria in Solid Tumors Table 1 OS was measured from the time of TACE to death or loss to follow‑up, while PFS was calculated from the time of TACE to primary and / or metastatic tumor progression.

### Statistical analysis 

This meta‑analysis was conducted using RevMan v5.3 (Cochrane Collaboration, Nordic Cochrane Centre, Copenhagen, Denmark) and Stata v12.0 (Stata Corporation, College Station, Texas, United States) software.

**Figure 1:**
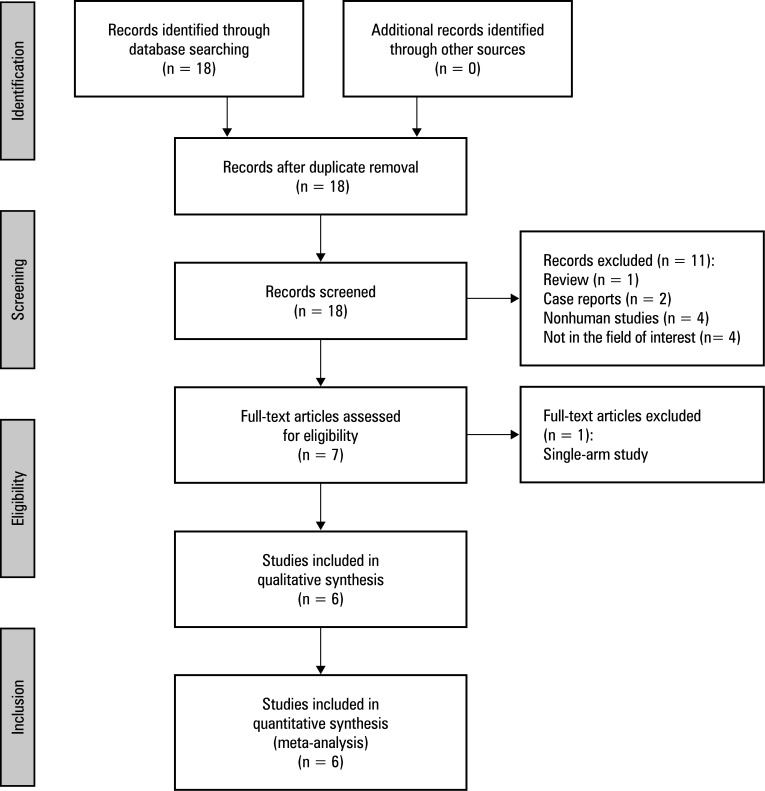
Meta‑analysis flow chart

**Table 2:** General characteristics of the included studies

Study	Year	Country / area	Design	NOS
Hu et al ^11^	2020	China	Retrospective	8
Hyder et al ^12^	2013	United States	Retrospective	6
Kuhlmann et al ^13^	2012	Germany	Retrospective	7
Sun et al ^14^	2022	China	Retrospective	8
Wang et al ^15^	2023	China	Randomized controlled trial	Not applicable
Zhang et al ^16^	2024	China	Retrospective	8

Abbreviations: NOS, Newcastle–Ottawa scale

Dichotomous variables were analyzed using pooled odds ratios (ORs) and 95% CIs. Hazard ratios (HRs) were used when assessing pooled OS and PFS rates. Heterogeneity was evaluated with the Q test and I^2^ statistic. In the case of significant heterogeneity (I^2^ >50%), data were analyzed with random‑effect models, otherwise, fixed‑effect models were employed. Sensitivity analyses were performed using the leave‑one‑out technique. A significance level of *P* below 0.05 was used as the threshold. The risk of publication bias was assessed using the Egger test.

### Ethics 

Approval of an ethics committee and written informed consent are not required for meta‑analyses.

## RESULTS 

### Study selection 

A total of 18 studies were found to be potentially relevant during the initial database search. Of those, 6 studies met the inclusion criteria.^11^^;^^12^^;^^13^^;^^14^^;^^15^^;^^16^ The study selection process is detailed in Figure 1.

**Figure 2:**
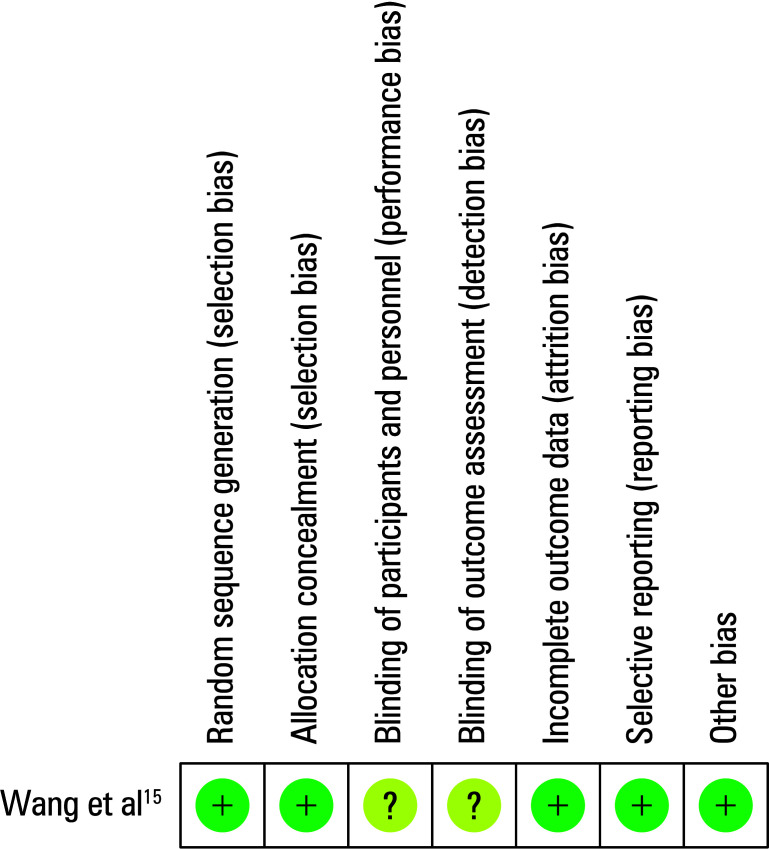
Quality assessment for the included randomized controlled trial.15 The green circles represent low risk of bias, while the yellow circles represent uncertain risk of bias.

Among the included studies, 5 were retrospective analyses and 1 was an RCT. General characteristics of all studies are presented in Table 2  Of the 5 retrospective studies, all exhibited NOS scores from 6 to 8. The RCT showed an uncertain risk of bias with respect to detection and performance Figure 2.

The studies enrolled 283 and 178 patients in the DEB‑TACE and cTACE groups, respectively. Patient characteristics are presented in Table 3 while the utilized chemical agents, OS data, and PFS data are detailed in Table 4.

### Objective response rate 

**Figure 3:**
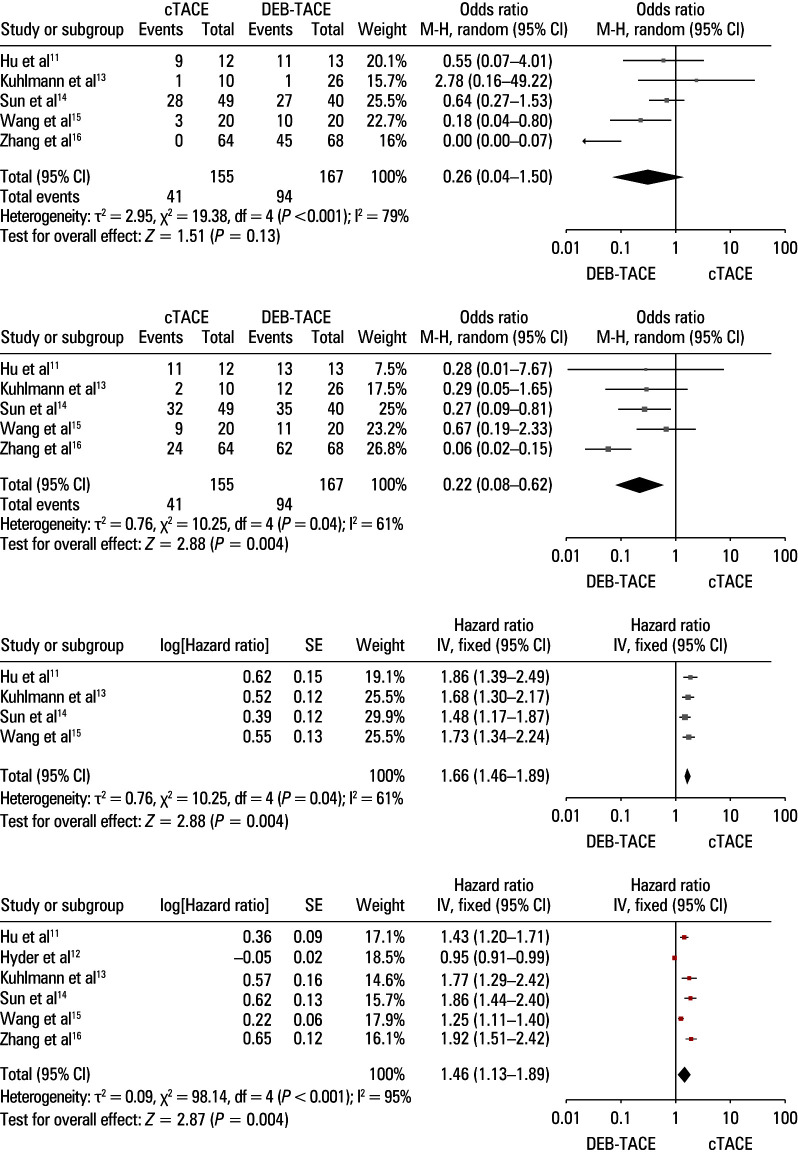
Pooled results for objective response rate (A), disease control rate (B), progression‑free survival (C), and overall survival (D) in the conventional and drug‑eluting bead transarterial chemoembolization groups Abbreviations: df, degree of freedom; IV, instrumental variable; MH, Mantel–Haenszel model; others, see Table 3

Data on ORR were provided in 5 studies enrolling 155 and 167 patients in the DEB‑TACE and cTACE groups, respectively.^11^^;^^13^^;^^14^^;^^15^In all studies, the ORR was analyzed within 3 months of the treatment. In the DEB‑TACE group, the ORR was higher than in the cTACE group (56.3% vs 26.5%; OR, 0.26; 95 CI%, 0.04–1.5;Figure 3**A**) but the difference was not significant. Substantial heterogeneity was observed (I^2^ = 79%); however, it was reduced when the study by Zhang et al16 was excluded from sensitivity analyses (I^2^ = 14%). Despite exclusion of this study, no substantial disparity in ORR was noted between the groups (*P* = 0.05).

**Table 3:** Characteristics of patients included in the analyzed studies

Study	Groups	Patients, n	Age, y	Sex (M/F)	Mean tumor size, mm	Tumor location	Child–Pugh classes	TNM stages	Number of tumors
Hu et al ^11^	cTACE	12	56.9	3/9	68	N/A	A–B	III–IV	≤3, 2 patients; >3, 10 patients
	DEB‑TACE	13	55.9	7/6	68	N/A	A–B	III–IV	≤3, 7 patients; >3, 6 patients
Hyder et al ^12^	cTACE	128	N/A	N/A	N/A	N/A	N/A	N/A	N/A
	DEB‑TACE	11	N/A	N/A	N/A	N/A	N/A	N/A	N/A
Kuhlmann et al ^13^	cTACE	10	62	8/2	N/A	N/A	N/A	N/A	N/A
	DEB‑TACE	26	67	15/11	N/A	N/A	N/A	N/A	N/A
Sun et al ^14^	cTACE	49	57.4	28/21	70	N/A	A–B	N/A	Single, 23 patients; multiple, 26 patients
	DEB‑TACE	40	61.8	25/15	79	N/A	A–B	N/A	Single, 16 patients; multiple, 24 patients
Wang et al ^15^	cTACE	20	N/A	11/9	N/A	N/A	A–B	III	N/A
	DEB‑TACE	20	N/A	17/3	N/A	N/A	A–B	III	N/A
Zhang et al ^16^	cTACE	64	56.9	46/18	84	Unilobar, 41 patients; bilobar, 23 patients	A–B	I–IV	≤4, 36 patients; >4, 28 patients
	DEB‑TACE	68	58.1	42/26	99	Unilobar, 44 patients; bilobar, 24 patients	A–B	I–IV	≤4, 36 patients; >4, 32 patients

Abbreviations: cTACE, conventional TACE; DEB, drug‑eluting bead; F, female; M, male; N/A, not available; TACE, transarterial chemoembolization; TNM, TNM Classification of Malignant Tumors

Wang et al^15^

### Disease control rate 

The DCR was reported in 3 studies^11^^;^^13^^;^^14^^;^^15^ that enrolled 155 and 167 patients in the DEB‑TACE and cTACE groups, respectively. In all these studies, the DCR was evaluated within 3 months of the treatment. The DEB‑TACE group exhibited a higher DCR than the cTACE group (79.6% vs 50.3%; OR, 0.22; 95 CI%, 0.08–0.62; *P* = 0.004; Figure 3**B**). While significant heterogeneity was evident (I^2^ = 61%), it disappeared once the study Zhang et al.^16^ was excluded from sensitivity analyses (I*^2^* = 0%). Even after exclusion of this study, DEB‑TACE was associated with a higher DCR than cTACE (*P* = 0.008).

### Progression‑free survival 

A total of 4 studies pro‑ vided data on PFS.^11^^;^^13^^;^^14^^;^^15^ The patients treated with DEB‑TACE presented longer PFS than those who underwent cTACE (HR, 1.66; 95% CI, 1.46–1.89; *P* <0.001; Figure 3**C**). There was no significant heterogeneity for this end point (I^2^ = 0%).

### Overall survival 

All studies reported on patient OS, with DEB‑TACE treatment being associated with longer OS than cTACE (HR, 1.46; 95% CI, 1.13–1.89; *P *= 0.004; Figure 3**D**). Significant heterogeneity was detected (I^2^ = 95%), but its source was not established through sensitivity testing.

### Adverse events 

Pooled AE incidence rates are reported in Table 5 Both groups exhibited similar rates of elevated ALT and AST levels, hyperbilirubinemia, and hypoalbuminemia (*P* = 0.59, *P *= 0.56, *P *= 0.64, and *P *= 0.23, respectively).

Figure description...

None of these AEs exhibited significant heterogeneity. However, heterogeneity testing could not be performed for the elevated AST end point owing to the 100% elevated AST rates in both groups in the study by Hu et al.^11^

### Publication bias 

Publication bias was only found to be significant for the OS outcome (Egger test, *P* = 0.002).

## DISCUSSION 

This meta‑analysis explored the efficacy and safety of cTACE and DEB‑TACE approaches for managing inoperable ICC based on patient therapeutic responses, AE occurrence, and survival rates. The results unequivocally showed that DEB‑TACE is superior to cTACE in terms of tumor control and survival outcomes.

Therapeutic response is the most important aspect of tumor treatment. In the current meta‑analysis, the DEB‑TACE group exhibited a higher ORR than the cTACE group (56.3% vs 26.5%). However, the difference was not significant (*P *= 0.13), even after adjusting for heterogeneity (*P* = 0.05). This may be related to the fact that ORR was evaluated at different time points across studies. In some of them, ORR was assessed at 1 to 2 months post‑treatment,^13^^;^^14^^;^^15^ while it has been suggested that DEB‑TACE requires at least 3 months to manifest its superior efficacy.^16^ ICCs are characterized by poorer blood supply than HCCs, potentially contributing to worse overall clinical efficacy of TACE‑based interventions in this patient population.

**Table 4:** Occurrence of adverse events

Study	Groups	Chemical agents	PFS, mo	OS, mo
Hu et al ^11^	cTACE	Gemcitabine and cisplatin	10.3	14
	DEB‑TACE	Gemcitabine and cisplatin	17	19.3
Hyder et al ^12^	cTACE	Gemcitabine, doxorubicin, mitomycin, and cisplatin	N/A	13.4
	DEB‑TACE	Doxorubicin and cisplatin	N/A	10.5
Kuhlmann et al ^13^	cTACE	Mitomycin	1.8	5.7
	DEB‑TACE	Irinotecan	3.9	11.7
Sun et al ^14^	cTACE	Epirubicin	2	6
	DEB‑TACE	Epirubicin	4	10
Wang et al ^15^	cTACE	Irinotecan	3	9
	DEB‑TACE	Irinotecan	8	19.5
Zhang et al ^16^	cTACE	Pirarubicin	N/A	5
	DEB‑TACE	Pirarubicin	N/A	12

Abbreviations: OS, overall survival; PFS, progression‑free survival; others see Table 3

**Table 5:** Occurrence of adverse events

Adverse event	Studies, n	OR (95% CI)	*P *value	Heterogeneity	Favor
Increased ALT level	2 ^11^**^;^**^16^	1.21 (0.61–2.43)	0.59	I^2 ^= 0%	–
Increased AST level	2 ^11^^;^^16^	1.24 (0.60–2.53)	0.56	Not applicable	–
Hyperbilirubinemia	3 ^11^^;^^15^^;^^16^	1.17 (0.61–2.25)	0.64	I^2^ = 0%	–
Hypoalbuminemia	2 ^11^^;^^16^	1.52 (0.77–3.02)	0.23	I^2^ = 7%	–

Abbreviations: ALT, alanine aminotransferase; AST, aspartate aminotransferase; OR, odds ratio

While no significant differences in pooled ORRs were noted in the present analysis, DEB‑TACE outperformed cTACE in terms of DCR. There are several possible explanations for this result. For one, cTACE employs lipiodol as an embolic material. This agent is highly fluid, with limited embolization activity. In contrast, DEBs are nondegradable, flexible, and deformable. Their use as an embolic material can facilitate long‑term embolization of tumor branches, triggering more extensive necrotic tumor cell death.^16^ Additionally, relative to the short‑acting nature of chemotherapeutic drugs delivered to the tumor during cTACE, utilization of DEBs can ensure persistent drug release. ^16^

We showed that DEB‑TACE was associated with superior OS and PFS outcomes, as compared with cTACE. This is in contrast with prior meta‑analyses focusing on the efficacy of DEB‑TACE in the management of HCC.^17^^;^^18^ The higher OS and PFS rates observed in the individuals treated with DEB‑TACE may be related to improved DCR in these patients. However, the OS end point was subject to significant heterogeneity, which may limit the reliability of our findings, emphasizing the need for additional, appropriately designed prospective RCTs to validate these results.

We found no discernible variations in the occurrence of AEs between the groups. As compared with the conventional approach, DEB‑TACE entails the delivery of higher doses of chemotherapeutic drugs and results in higher local concentrations of these drugs in the target tumor, which may increase the risk of AE occurrence.^19^ However, the ability of DEBs to mediate local drug release may be helpful in protecting against systemic toxicity.^20^^;^^21^

At present, radioactive stent insertion (RSI) is widely used for treating hilar cholangiocarcinoma's.^22^ The radioactive stent can both relieve jaundice and deliver brachytherapy to the tumor. However, ICC is a mass‑like tumor; therefore, RSI is not a suitable treatment option.

The current meta‑analysis has certain limitations. Firstly, most of the included studies were retrospective, which is associated with increased selection bias. Secondly, unbalanced patient numbers were evident for some of the studies, potentially leading to worsening of any selection bias. Lastly, treatment responses were assessed at different time points in the included studies, potentially leading to further bias.

## CONCLUSIONS 

In summary, both chemoembolization strategies exhibited similar safety profiles. However, DEB‑TACE outperformed cTACE in terms of therapeutic efficacy, highlighting the potential for broader application of DEB‑TACE in the management of patients with inoperable ICC.

## References

[BIBR-1] Sun J.J., Qian X.L., Shi Y.B. (2023). Clinical and magnetic resonance imaging features predict microvascular invasion in intrahepatic cholangiocarcinoma. Prz Gastroenterol.

[BIBR-2] Ma X., Qian X., Wang Q. (2023). Radiomics nomogram based on optimal VOI of multi-sequence MRI for predicting microvascular invasion in intrahepatic cholangiocarcinoma. Radiol Med.

[BIBR-3] Chen S., Wan L., Zhao R. (2023). Predictive factors of microvascular invasion in patients with intrahepatic mass-forming cholangiocarcinoma based on magnetic resonance images. Abdom Radiol (NY.

[BIBR-4] Guglielmi A., Ruzzenente A., Campagnaro T. (2009). Intrahepatic cholangiocarcinoma: prognostic factors after surgical resection. World J Surg.

[BIBR-5] Tsukamoto M., Yamashita Y.I., Imai K. (2017). Predictors of cure of intrahepatic cholangiocarcinoma after hepatic resection. Anticancer Res.

[BIBR-6] He M., Jiang N., Yin X. (2023). Conventional and drug-eluting beads transarterial chemoembolization in patients with unresectable intrahepatic cholangiocarcinoma: a systematic review and pooled analysis. J Cancer Res Clin Oncol.

[BIBR-7] Liu D., Wang J., Ma Z. (2022). Treatment of unresectable intrahepatic cholangiocarcinoma using transarterial chemoembolisation with irinotecan-eluting beads: analysis of efficacy and safety. Cardiovasc Intervent Radiol.

[BIBR-8] Zhang H., Han C., Zheng X. (2023). Significant response to transarterial chemoembolization combined with PD-1 inhibitor and apatinib for advanced intrahepatic cholangiocarcinoma: a case report and literature review. J Cancer Res Ther.

[BIBR-9] Dhanasekaran R., Kooby D.A., Staley C.A. (2010). Comparison of conventional transarterial chemoembolization (TACE) and chemoembolization with doxorubicin drug eluting beads (DEB) for unresectable hepatocelluar carcinoma (HCC. J Surg Oncol.

[BIBR-10] Cook D.A., Reed D.A. (2015). Appraising the quality of medical education research methods: the Medical Education Research Study Quality Instrument and the Newcastle-Ottawa Scale-Education. Acad Med.

[BIBR-11] Hu Y., Hao M., Chen Q. (2020). Comparison of the efficacy and safety among apatinib plus drug-eluting bead transarterial chemoembolization (TACE), apatinib plus conventional TACE and apatinib alone in advanced intrahepatic cholangiocarcinoma. Am J Transl Res.

[BIBR-12] Hyder O., Marsh J.W., Salem R. (2013). Intra-arterial therapy for advanced intrahepatic cholangiocarcinoma: a multi-institutional analysis. Ann Surg Oncol.

[BIBR-13] Kuhlmann J.B., Euringer W., Spangenberg H.C. (2012). Treatment of unresectable cholangiocarcinoma: conventional transarterial chemoembolization compared with drug eluting bead-transarterial chemoembolization and systemic chemotherapy. Eur J Gastroenterol Hepatol.

[BIBR-14] Sun T., Zhang W., Chen L. (2022). A comparative study of efficacy and safety of transarterial chemoembolization with CalliSpheres and conventional transarterial chemoembolization in treating unresectable intrahepatic cholangiocarcinoma patients. J Cancer.

[BIBR-15] Wang J., Xue Y., Liu R. (2023). DEB-TACE with irinotecan versus C-TACE for unresectable intrahepatic cholangiocarcinoma: a prospective clinical study. Front Bioeng Biotechnol.

[BIBR-16] Zhang Z., Jiang N., Yin X. (2024). Comparison of efficacy and safety of conventional transarterial chemoembolization and drug-eluting bead transarterial chemoembolization in unresectable intrahepatic cholangiocarcinoma: a multicenter retrospective cohort study. Eur J Radiol.

[BIBR-17] Zou J.H., Zhang L., Ren Z.G., Ye S.L. (2016). Efficacy and safety of cTACE versus DEB-TACE in patients with hepatocellular carcinoma: a meta-analysis. J Dig Dis.

[BIBR-18] Ayyub J., Dabhi K.N., Gohil N.V. (2023). Evaluation of the safety and efficacy of conventional transarterial chemoembolization (cTACE) and drug-eluting bead (DEB)-TACE in the management of unresectable hepatocellular carcinoma: a systematic review. Cureus.

[BIBR-19] Lee S., Kim K.M., Lee S.J. (2017). Hepatic arterial damage after transarterial chemoembolization for the treatment of hepatocellular carcinoma: comparison of drug-eluting bead and conventional chemoembolization in a retrospective controlled study. Acta Radiol.

[BIBR-20] Savic L.J., Chapiro J., Geschwind J.H. (2017). Intra-arterial embolotherapy for intrahepatic cholangiocarcinoma: update and future prospects. Hepatobiliary Surg Nutr.

[BIBR-21] Facciorusso A. (2018). Drug-eluting beads transarterial chemoembolization for hepatocellular carcinoma: Current state of the art. World J Gastroenterol.

[BIBR-22] Zhang Y., Wang P., Xu G., Chen M. (2022). Bilateral versus unilateral radioactive stent insertion for hilar cholangiocarcinoma. Wideochir Inne Tech Maloinwazyjne.

